# Genomic Heterogeneity in a Natural Archaeal Population Suggests a Model of tRNA Gene Disruption

**DOI:** 10.1371/journal.pone.0032504

**Published:** 2012-03-05

**Authors:** Junichi Sugahara, Kosuke Fujishima, Takuro Nunoura, Yoshihiro Takaki, Hideto Takami, Ken Takai, Masaru Tomita, Akio Kanai

**Affiliations:** 1 Institute for Advanced Biosciences, Keio University, Tsuruoka, Japan; 2 Systems Biology Program, Graduate School of Media and Governance, Keio University, Fujisawa, Japan; 3 Subsurface Geobiology & Advanced Research Project, Extremobiosphere Research Program, Institute of Biogeosciences, Japan Agency for Marine–Earth Science & Technology, Yokosuka, Japan; 4 Microbial Genome Research Group, Extremobiosphere Research Program, Institute of Biogeosciences, Japan Agency for Marine–Earth Science & Technology, Yokosuka, Japan; Max-Planck-Institute for Terrestrial Microbiology, Germany

## Abstract

Understanding the mechanistic basis of the disruption of tRNA genes, as manifested in the intron-containing and split tRNAs found in *Archaea*, will provide considerable insight into the evolution of the tRNA molecule. However, the evolutionary processes underlying these disruptions have not yet been identified. Previously, a composite genome of the deep-branching archaeon *Caldiarchaeum subterraneum* was reconstructed from a community genomic library prepared from a *C. subterraneum*–dominated microbial mat. Here, exploration of tRNA genes from the library reveals that there are at least three types of heterogeneity at the tRNA^Thr^(GGU) gene locus in the *Caldiarchaeum* population. All three involve intronic gain and splitting of the tRNA gene. Of two fosmid clones found that encode tRNA^Thr^(GGU), one (tRNA^Thr-I^) contains a single intron, whereas another (tRNA^Thr-II^) contains two introns. Notably, in the clone possessing tRNA^Thr-II^, a 5′ fragment of the tRNA^Thr-I^ (tRNA^Thr-F^) gene was observed 1.8-kb upstream of tRNA^Thr-II^. The composite genome contains both tRNA^Thr-II^ and tRNA^Thr-F^, although the loci are >500 kb apart. Given that the 1.8-kb sequence flanked by tRNA^Thr-F^ and tRNA^Thr-II^ is predicted to encode a DNA recombinase and occurs in six regions of the composite genome, it may be a transposable element. Furthermore, its dinucleotide composition is most similar to that of the pNOB8-type plasmid, which is known to integrate into archaeal tRNA genes. Based on these results, we propose that the gain of the tRNA intron and the scattering of the tRNA fragment occurred within a short time frame *via* the integration and recombination of a mobile genetic element.

## Introduction

Transfer RNA (tRNA) is a small RNA molecule that plays a key role in protein biosynthesis. A cloverleaf secondary structure and L-shaped tertiary structure are well-known features of mature tRNAs that are strictly conserved across all three domains of life: *Archaea*, *Bacteria*, and *Eukarya* [1], [2]. However, diverse arrangements of the genes encoding tRNAs have been reported. In these cases, the tRNA genes are never directly transcribed into a contiguous tRNA but instead require processing by splicing to form the standard tRNA structure. Interruption by a single intron is the most common cause of tRNA-gene disruption in archaeal and eukaryotic genomes [3], [4]. Although the tRNA intron is most often inserted between nucleotides 37 and 38 (37/38) of the tRNA gene, tRNA genes containing non-canonical introns in various positions have recently been reported [5], as have tRNA genes containing as many as three introns [5], [6], [7]. In Archaea, a more unusual tRNA arrangement, the so-called ‘split tRNA’, has been reported. This is produced by the *trans* splicing of two or three pieces of RNA transcribed from different genes [8], [9], [10]. Each member of the split-tRNA pair contains a flanking leader sequence at either the 5′ or 3′ end, and these form complementary RNA sequences that hybridize in the cell. The intron-containing tRNA and the split tRNA share a common structural motif, called the ‘bulge–helix–bulge’, at the intron/leader–exon boundary. This motif is recognized by tRNA splicing endonucleases, suggesting that the intron-containing tRNA and the split tRNA are evolutionarily related [8], [9], [10], [11], [12], [13].

The discovery of disrupted tRNA genes has introduced the challenge of understanding when and why these complex processing pathways for tRNA emerged, and how they have affected tRNA gene evolution. In *Archaea*, many mobile genetic elements, such as conjugative plasmids and viruses, have been reported to integrate into the host genome *via* site-specific recombination events that occur at short homologous regions. Owing to their highly conserved nature, tRNA genes are frequent target sites for integration [14], [15], [16]. Randau and Söll proposed that integration events are one of the possible evolutionary pressure causing the disruption of tRNA to exist as a disrupted gene [17], and argued that intron gain and tRNA fragmentation will prevent recombination if the disruption occurs at the attachment site for viral or plasmid DNA in the host chromosome. Such archaeal mobile elements have been predominantly isolated from geothermal environments [15], [18], [19], which are also known as the habitats of tRNA-intron-rich members of the archaeal phylum *Crenarchaeota*. However, there is no direct evidence of an evolutionary relationship between disrupted tRNA genes and the integration of mobile elements.

Previously, a metagenomic (community genomic) library consisting of 5,280 fosmid clones was constructed from a Hot Water Crenarchaeotic Group I (HWCGI)-dominated microbial-mat community collected from a geothermal water stream in a subsurface Japanese gold mine [20], [21]. The HWCGI comprises putative thermophiles known to occupy a relatively deep position within the crenarchaeotic lineages, and could constitute the candidate division *Aigarchaerota* [20], [21], [22]. From the library, we reconstructed a composite genome for the uncultivated archaeon Candidatus *Caldiarchaeum subterraneum* (∼1.7 Mbp), a member of the HWCGI (*Aigarchaeota*). We also reported 45 nonredundant tRNA genes, which can decode all sense codons in the composite genome [21]. Among these 45 tRNA genes, 13 are predicted to be intron-containing tRNAs, and three of these 13 contain multiple introns (tRNA^Leu^[UAA], tRNA^Glu^[CUG], and tRNA^Thr^[GGU]). The tRNA introns are located not only at the canonical position 37/38, but also at various noncanonical positions, such as the D-arm, V-arm, and T-arm of the tRNA, as has been observed in other crenarchaeal species [7]. This suggests that *C. subterraneum* is one of the tRNA-intron-rich archaea.

We further explored the tRNA genes from the community genomic library to deepen our understanding of tRNA gene disruption during evolution. The library is reported to contain heterogeneous genomic fragments of *C. subterraneum*, which were used for assembly of the composite genome [21]. A comparison of these heterogeneous genomic fragments and the composite genome revealed that there are at least three types of heterogeneity at the tRNA^Thr^(GGU) gene locus in the *Caldiarchaeum* population, all of which involve intron gain and splitting of the tRNA gene. We propose that this heterogeneity was generated by the integration of mobile genetic elements. Here, based on sequence evidence observed at the strain or sub-strain level in a natural microbial community, we present a model describing a possible mechanism responsible for the disruption of tRNA genes.

## Results

### Occurrence of two types of tRNA^Thr^(GGU) genes and a tRNA^Thr^(GGU) fragment in a *C. subterraneum* population

**Figure 1:**
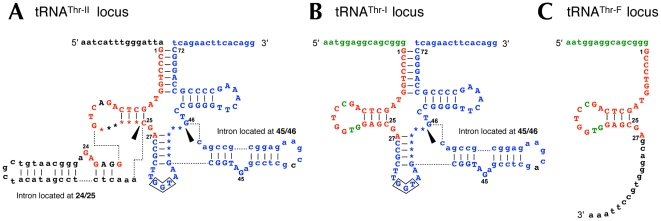
Sequences and secondary structures of the DNA regions encoding the tRNA^Thr^(GGU) genes and a tRNA^Thr^(GGU) fragment. (A) tRNA^Thr^ (GGU) gene locus with two introns (tRNA^Thr-II^) found in the composite genome of *C. subterraneum*. (B) tRNA^Thr^(GGU) gene locus with one intron (tRNA^Thr-I^) found in clone JFF006_G04. (C) tRNA^Thr^(GGU) fragment (tRNA^Thr-F^) found in the composite genome. Positions in the tRNA are numbered as described previously [2]. Nucleotides in the tRNA exon are shown in upper-case letters. Nucleotides conserved among A, B, and C, between A and B, and between B and C are shown as red, blue, and green, respectively. Black triangles indicate the locations of introns. The predicted anticodon is boxed. The 15-nt extensions at the 5′ and 3′ termini of the tRNA structures are defined randomly, and are not the actual leader or trailer sequences of potential pre-tRNAs.

Recently, we reported a tRNA^Thr^(GGU) gene containing two introns at nucleotide positions 24/25 and 45/46 (Figure 1A) in a composite genome of *C. subterraneum* that was reconstructed from a community genomic library prepared from a *C. subterraneum*–dominated microbial-mat community [21]. During the reconstruction of the composite genome, we found another tRNA^Thr^(GGU) gene containing only one intron at position 45/46 (Figure 1B) in fosmid clone JFF006_G04. To distinguish these two types of sequences hereafter, we defined the tRNA^Thr^(GGU) gene containing two introns found in the composite genome as ‘tRNA^Thr-II^’, and the other as ‘tRNA^Thr-I^’, reflecting the number of introns they contain. Although we did not identify the tRNA^Thr-I^ sequence in the composite genome of *C. subterraneum* following a homology search, we did find a sequence fragment identical to the 5′ half of tRNA^Thr-I^ (tRNA^Thr-F^; Figure 1C). In Figure 1, the nucleotide sequences of tRNA^Thr-II^, tRNA^Thr-I^, and tRNA^Thr-F^ are shown with their upstream and downstream genomic regions. Sequence comparison revealed that the sequence of the 5′ region of tRNA^Thr-I^ (nucleotide positions 1–27) and its upstream sequence are identical to those of the corresponding regions in tRNA^Thr-F^. The 3′ region of tRNA^Thr-I^ (nucleotide positions 25–72) and its downstream sequence are identical to those of the corresponding regions in tRNA^Thr-II^, except for a single-nucleotide difference in the intron region. These observations strongly suggest that tRNA^Thr-I^ is a fusion of tRNA^Thr-F^ and tRNA^Thr-II^. We also confirmed that, whereas the nucleotide sequence of the 5′ region of tRNA^Thr-F^ is identical to that of the corersponding region of tRNA^Thr-I^(GGU), there are three single-nucleotide mismatches between both of these sequences and the corresponding 5′ regions in either tRNA^Thr^(UGU) or tRNA^Thr^(CGU).

**Figure 2:**
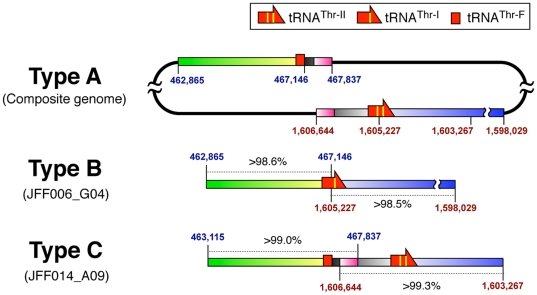
Genomic heterogeneity in tRNA^Thr^(GGU) gene loci in the composite genome and cloned sequences. Schematic representation of the composite genome of *C. subterraneum* and the nucleotide sequences of clones JFF006_G04 and JFF014_A09 are shown (types A, B, and C, respectively). Homologous regions between the composite genome and the cloned sequences are shown in the same colors, and the similarity scores (%) against the corresponding regions in the composite genome are indicated on the cloned sequences. The genomic positions of the two distantly located regions in the composite genome are shown as red and blue numbers.

We further analyzed the nucleotide sequence of the JFF006_G04 clone to clarify the evolutionary relationships between tRNA^Thr-II^, tRNA^Thr-I^, and tRNA^Thr-F^. Intriguingly, we found that clone JFF006_G04 is a hybrid sequence consisting of two distantly located regions in the composite genome of *C. subterraneum*, and that pairwise matches with these regions show over 98.6% sequence similarity in a 4.3 kb stretch upstream of the tRNA^Thr-I^ gene and over 98.5% sequence similarity in a 7.2 kb region downstream from the tRNA^Thr-I^ gene (Figure 2). The high sequence similarity extending over 10 kb of the composite genome strongly suggests that the clone was derived from a *C. subterraneum* strain or at least from a closely related (sub-)species. Moreover, because almost all archaeal chromosomes encode nonredundant tRNA genes [23], the presence of tRNA^Thr-II^ and tRNA^Thr-I^ demonstrates the existence of heterogeneity in the tRNA genes in the *Caldiarchaeum* population. Therefore, we defined the heterogeneous tRNA^Thr^(GGU) gene loci found in the composite genome and in JFF006_G04 as type A and type B, respectively (Figure 2).

To clarify whether other heterogeneous sequences encoding the tRNA^Thr^ gene exist in the *Caldiarchaeum* population, we collected additional fosmid clones that represent the tRNA^Thr^(GGU) gene locus in the genomic library and determined their sequences. In this way, we identified another heterogeneous fosmid clone, JFF014_A09 (defined as type C), which contains two distinct genomic regions of the composite genome, with over 99.0% and 99.3% sequence similarity (Figure 2). Although clone JFF014_A09 harbors a region identical to that found in clone JFF006_G04, the tRNA^Thr-I^ gene is not present in clone JFF014_A09. Instead, tRNA^Thr-F^ and tRNA^Thr-II^, which are distantly located in the composite genome, are found close to one another in clone JFF014_A09, being separated by an interval of only approximately 1,800 bp (shown as black and pink gradations in Figure 2). In clone JFF014_A09, a 545-bp sequence (shown as a pink gradated region in Figure 2) was mapped to both of the distinct tRNA^Thr^ loci in the composite genome (Figure 2), suggesting that this overlapping sequence may be a recombination region. To avoid misinterpretation caused by cloning artifacts, we obtained and analyzed at least two independent fosmid clones for each tRNA type (Table S1). Based on the results, we concluded that the proposed genome structures segregated individually in the microbial population.

### Characterization of the insertion sequence at the tRNA^Thr^(GGU) gene locus

**Figure 3:**
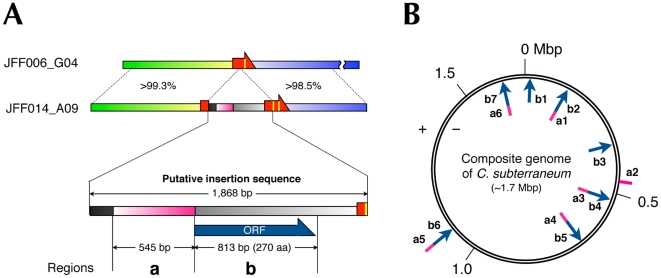
Characterization of the insertion sequence found at the tRNA^Thr^ gene locus. (A) Schematic representations of clones JFF006_G04 and JFF014_A09, and the enlarged putative insertion sequence. The colors correspond to those used in Figure 2. Homologous regions between the cloned sequences are linked by a dashed line and the similarity scores are shown. The two regions ‘a’ (pink) and ‘b’ (blue arrow) are shown, along with their respective lengths. (B) Regions homologous to the ‘a’ and ‘b’ sequences (at the nucleotide level for ‘a’ and amino acid level for ‘b’) are mapped to the schematic representation of the composite genome, and each is numbered (a1–a6 or b1–b7).

Because clone JFF014_A09 contains a putative recombination region that separates tRNA^Thr-II^ and tRNA^Thr-F^, this clone provides a valuable key to understanding how genomic heterogeneity was generated around the tRNA^Thr^(GGU) gene locus in the *Caldiarchaeum* population. As shown in Figure 3A, a comparison of clones JFF006_G04 and JFF014_A09 revealed that a 1,868-bp sequence extending from the end of tRNA^Thr-F^ to nucleotide position 27 of tRNA^Thr-II^ (including the intron located at positions 24/25) in clone JFF014_A09 is an insertion sequence. As described above, the putative recombination region (545 bp) is included in this insertion sequence (defined as region ‘a’). We also found an open reading frame (ORF) of 813 bp (defined as region ‘b’) encoded immediately downstream of region ‘a’ (Figure 3A). Interestingly, protein family analysis using SVMProt software [24] suggested that the ‘b’ protein belongs to families of proteins that function in DNA-binding and/or DNA recombination, with an 88.1%–89.3% probability of correct classification of protein function.

A homology search of regions ‘a’ and ‘b’ (a nucleotide search for region ‘a’ and an amino acid search for region ‘b’) against the composite genome of *C. subterraneum* revealed the existence of several similar nucleotide and amino acid sequences (Figure 3B). In the composite genome, six regions (a1–a6) show pairwise matches to region ‘a’ with an *E*-value<10^−100^, with over 80% coverage (Figure S1). In contrast, no significant alignment of region ‘b’ was detectable at the nucleotide level, although seven (b1–b7) protein sequences from among the 1,793 ORFs in the composite genome showed pairwise matches to the ‘b’ protein with an E-value<10^−20^, with over 80% coverage (Figure S2). Interestingly, five of these seven ‘b’ regions were coupled to an ‘a’ region (Figure 3B). These results suggest that the genomic region consisting of ‘a’ and ‘b’ sequences is a transposable element. Furthermore, we analyzed the genes flanking the insertion sequences and found that the 5′ upstream sequence (nucleotide positions 503442–503515) of the a3b4 region (Figure 3B) encodes another tRNA^Thr^, although most of the other flanking genes turned out to encode hypothetical proteins. This tRNA^Thr^ contains a CGU anticodon and no intronic sequence in its gene. However, there is no tRNA^Thr^(CGU) fragment in the composite genome. Based on the nucleotide comparisons, it is possible to distinguish fragmented tRNA^Thr^(GGU) from tRNA^Thr^(CGU). This finding suggests that among tRNAs, tRNA^Thr^ may be a specific target for insertion sequences.

We also conducted BLAST analysis against all fosmid clones to see whether similar genomic alterations have occurred in other ‘a’ and ‘b’ regions. The analysis showed no such alterations, except at tRNA^Thr^ loci (data not shown). In other words, tRNA^Thr^ loci are the only loci that show these types of frequent genomic alterations. We also analyzed all tRNA loci in the *C. subterraneum* fosmid clones and found again that tRNA^Thr^ is the only tRNA that shows this type of genomic heterogeneity. Based on previous reports of tRNA-specific recombination by viruses and plasmids [14], [25], we propose that recombination driven by these agents may also be responsible for archaeal tRNA^Thr^-specific recombination.

**Figure 4:**
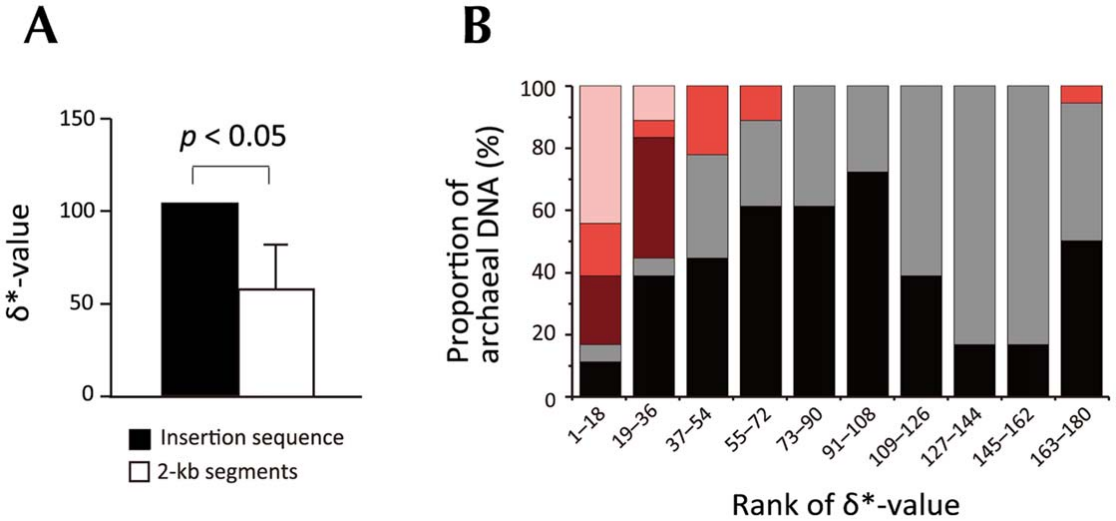
Dinucleotide bias analysis of the insertion sequence. (A) The average absolute dinucleotide relative abundance difference (δ*-value) for the composite genome and the insertion sequence (1,868 bp) was compared with the δ*-values for the composite genome and each of the computationally generated 2-kb genomic segments (*n* = 1,681). An error bar represents the standard deviation. Statistical significance was determined by calculating the proportion of δ*-values for the 2-kb segments that were greater than or equal to that of the insertion sequence. (B) Relative abundances of various types of archaeal DNA sequences, ranked according to the similarity of their dinucleotide content to the insertion sequence. In total, 180 DNA sequences were sorted in ascending order of their δ*-values and divided into 10 bins. The sequences in each bin were categorized into five groups: plasmids encoding pNOB8-type integrases (pink, *n* = 10); plasmids in the order *Sulfolobales* (red, *n* = 11); chromosomes in the order *Sulfolobales* (dark red, *n* = 11); other plasmids (dark grey, *n* = 74), and other chromosomes (black, *n* = 74). See Table S2 for the complete list of all 180 DNAs.

Next, we performed dinucleotide bias analysis [26] to further determine the characteristics of the insertion sequence. It has previously been reported that the dinucleotide compositional profile is relatively constant throughout the genome, except in regions that were recently acquired *via* horizontal transfer [26], [27]. Therefore, we calculated the average absolute dinucleotide relative abundance difference (the so-called ‘δ*-value’, which indicates the difference in the dinucleotide compositions of two DNA sequences) between the whole composite genome of *C. subterraneum* and the insertion sequence found in clone JFF014_A09 (Figure 4A). The δ*-value showed a significantly higher score than the average of the δ*-values between the composite genome and each of the computationally generated 2-kb genomic segments spanning the composite genome with 1-kb overlaps (Figure 4A). Given that a high δ*-value indicates a large difference in the dinucleotide compositions of two sequences, this result suggests that the insertion sequence was acquired recently.

If this speculation is true, then it introduces questions concerning the origin of this sequence. To investigate this, we calculated the δ*-values between the insertion sequence and each of 180 various types of DNA sequences, including 85 archaeal chromosomes and 95 archaeal plasmids (Table S2). Many conjugative plasmids that integrate into the tRNA genes of the host genome have been reported from members of the archaeal order *Sulfolobales* [15], [28]. They usually encode a ‘pNOB8-type’ integrase that acts as a recombinase. Therefore, we initially identified the plasmids encoding pNOB8-type integrases from 95 archaeal plasmids, and then observed the relative distributions of their δ*-values (Figure 4B). In this way, we identified 10 plasmids encoding pNOB8-type integrases, all of which occur in the order *Sulfolobales*. Surprisingly, eight of these 10 plasmids were ranked in the top 18 for δ*-values among the 180 DNAs, so that they constituted more than 40% of the top 10% bin in Figure 4B. Although other types of DNA sequences that occur in the *Sulfolobales* also showed a clear bias towards higher ranking, the pNOB8-type plasmids all showed lower ranking than the other *Sulfolobales* DNAs (Figure 4B). We also analyzed the genomes of archaeal viruses known to target the tRNA genes and a provirus-like sequence that is found in the composite genome of *C. subterraneum*. However, there was no significant bias in their δ*-values when compared with the insertion sequence (data not shown). This suggests that the insertion sequence has been laterally transferred from a plasmid encoding a pNOB8-type integrase, and previously found in the *Sulfolobales* crenarchaeotes. However, the pH in our environmental sample was close to neutral and the environment was quite different from that of *Sulfolobales*, which lives under acidic conditions. Therefore, direct interaction between *C. subterraneum* and *Sulfolobales* seems unlikely. We also considered the possibility that pNOB-type plasmids or related plasmids are widely distributed among species. However, there is little information to support or contradict this notion because the plasmids that have been analyzed are mainly derived from members of *Sulfolobales*. In addition, the plasmid pGT5 from *Pyrococcus abyssi* GE5 was ranked second out of 180 DNAs in the δ*-value analysis. Thus, we suggest that more archaeal plasmid sequences are required to address the exact plasmid family that harbors the insertion sequences in the *Caldiarchaeum* genome.

## Discussion

**Figure 5:**
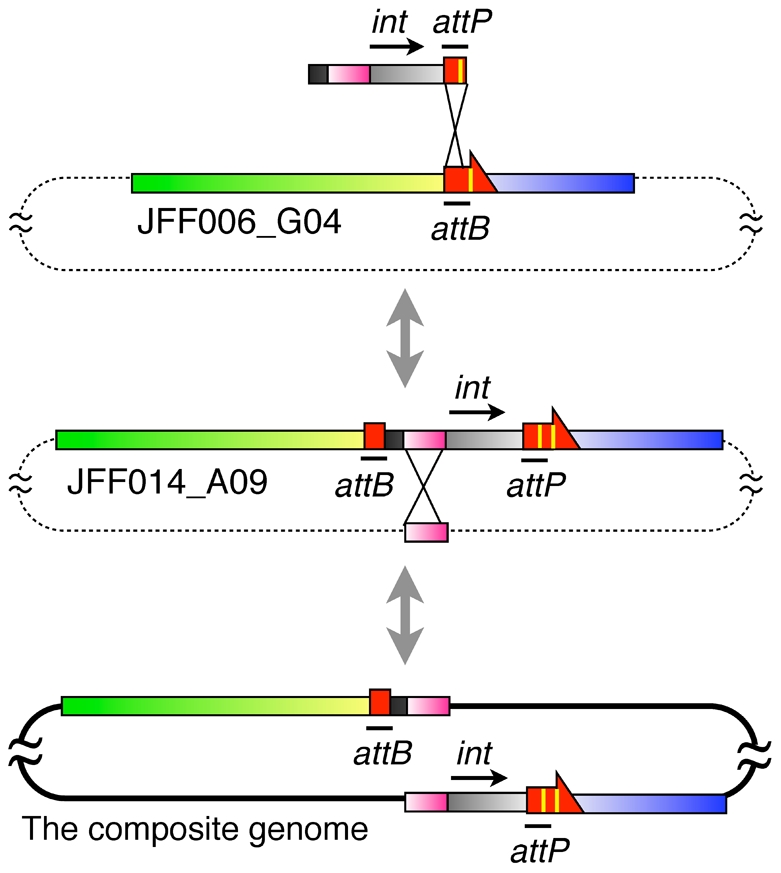
Model explaining the intron gain and fragmentation of the tRNA gene. A model for the intron gain and fragmentation processes of tRNA genes among the heterogeneous *C. subterraneum* genomes through site-specific recombination of a mobile element and homologous recombination. Dotted lines indicate the genomes of putative heterogeneous *C. subterraneum* strains. The tRNA^Thr^ gene, a fragment of it, and its intron are denoted by a red arrow, red rectangle, and yellow line, respectively (not to scale). The colors correspond to those in Figure 2. Whereas *int* represents the integrase gene, *attP* and *attB* denote the attachment sites for integration.

In this study, we identified genomic heterogeneity at the tRNA^Thr^(GGU) gene locus in a naturally occurring *Caldiarchaeum* population. This observation led us to consider the process by which disrupted tRNA genes, including multiple-intron-containing tRNAs [5], [6], [7] and split tRNAs [8], [9], [10], are generated in the domain *Archaea*. To examine the mechanism of tRNA gene disruption, we formulated a model explaining the evolutionary relationships among clones encoding the tRNA^Thr^(GGU) gene locus identified in this study (Figure 5). As described above, we found heterogeneous sequences at the tRNA^Thr^(GGU) gene locus, and some of the cloned fragments consisted of two distantly located regions in the composite *C. subterraneum* genome (Figure 2). Moreover, the similarities between these sequences and the corresponding regions in the composite genome were greater than 98%, even though the pairwise matches extended over 11,000 bp. This strongly suggests that the sequences of the clones are derived from the genome of *C. subterraneum* or closely related (sub-)species. Of the 21 16S rRNA gene sequences we isolated from a metagenomic library prepared from a naturally occurring *Caldiarchaeum* population, only two of them were designated as ‘*Caldiarchaeum* type II’. Whereas these two exhibit 96.6% similarity to the *C. subterraneum* sequence, no mismatch residues were found among the remaining 19 of *C. subterraneum* 16S rRNA gene exon sequences [21]. Given the higher similarity of homologous regions found in the tRNA^Thr^ loci (>98.5%) compared to the similarity between the 16S rRNA genes of *C. subterranuem* and *Caldiarchaeum* type II (96.6%), the abundances of three tRNA^Thr^ locus types are inconsistent with the abundance of clones of the *Caldiarchaeum* type II 16S rRNA gene in the metagenomic library. Furthermore, based on the typical criteria for species classification [29], [30], we speculate that the same species can have heterogeneous tRNA genomic loci. Nonetheless, we cannot eliminate the possibility, however slight, that the heterogeneous tRNAs are derived from distinct but extremely closely related species. Finally, because most archaeal chromosomes encode nonredundant tRNA genes [23], the presence of these clones indicates that at least three types of heterogeneous chromosomes exist separately in the *C. subterraneum* or closely related (sub-)species that harbor same 16S rRNA gene exon sequences (Figure 5). This also suggests that intron gain or loss in tRNA genes and the scattering of tRNA fragments to remote parts of the genome possibly occurred over a short time period within a single species, or among two extremely closely related species during evolution.

How might these heterogeneities have arisen? We considered that the integration of a mobile genetic element might have been a key factor in this phenomenon. In *Archaea*, many mobile elements, such as conjugative plasmids and viruses, have been reported to integrate into the host chromosome by site-specific recombination This integration occurs within short homologous regions between the host DNA (known as *attB* sites) and *attP* sites within the plasmid or viral DNA [17]. Intriguingly, the 5′ or 3′ terminus of the tRNA sequence is the typical attachment site. For example, the *attP* site of the *Sulfolobus* spindle virus SSV1 is a 44-bp sequence homologous to the 3′ terminus of the host tRNA^Arg^ gene [14], [25]. If the virus integrates into the tRNA^Arg^ gene, a reconstituted tRNA^Arg^ gene and a 44-bp direct repeat of the tRNA^Arg^ gene are generated at the integration borders of the SSV1 provirus. In such processes, a plasmid- or virus-encoded integrase acts as a site-specific recombinase [15]. Archaeal integrases have previously been classified as being of either the SSV-type or the pNOB8-type [31]. In a mobile element that encodes an SSV-type integrase (mostly found in viruses), the sequence of the *attP* site is included in the gene encoding the integrase. Therefore, upon its integration, the integrase gene is partitioned into two sequences, each encoding either the N- or C-terminal portion of the integrase, which overlap with the reconstituted tRNA gene and the direct repeat [14], [25]. In contrast, because the gene encoding the pNOB8-type integrase (mostly encoded on plasmids) separates the *attP* site, the host genome contains an intact integrase gene located immediately adjacent to the reconstituted tRNA gene on the same strand [15]. We noted that the integration borders of pNOB8-type plasmids bear a striking resemblance to this feature of JFF014_A09. First, in clone JFF0014_A09, tRNA^Thr-F^, which is homologous to the 5′ terminus of the tRNA^Thr^(GGU) sequence, is located upstream of the tRNA^Thr-II^ gene, forming a direct repeat of the tRNA (Figure 2). Second, a comparison of clones JFF014_A09 and JFF006_G04 revealed an insertion sequence that constitutes a genomic region flanked by direct repeats of the tRNA sequence (Figure 3A). Third, in the insertion sequence, region ‘b’, which encodes a putative DNA recombinase, is immediately adjacent to the tRNA^Thr-II^ gene on the same strand. Therefore, because the sequence encoding the putative DNA recombinase does not overlap with the tRNA sequences, we deduced that the insertion sequence in JFF014_A09 might be derived from a pNOB8-type plasmid. This speculation is further supported by the observation that the dinucleotide composition of the insertion sequence is similar to that of some conjugative plasmids encoding pNOB8-type integrases (Figure 4B).

We observed the gain of an extra intron at position 24/25 of the tRNA^Thr^(GGU) gene in the *C. subterraneum* genome. Recently, it has been reported that base mismatches between the *attP* site and *attB* site are inherited in reconstituted tRNA genes [16], [17]. This suggests that the recombination of two similar sequences, one of which includes a redundant sequence such as a tRNA intron, may occur during the integration of a mobile element. Our study confirms this possibility, in that the tRNA intron was derived from a mobile element that integrated *via* site-specific recombination between an *attP* sequence containing an intron and an *attB* sequence that did not contain an intron (Figure 5). In the host archaeal genome, the gain of an intron in the *attB* site of tRNA gene might be a protective mechanism against the invasion of conjugative plasmids and viruses [17]. The genomic heterogeneity in the archaeal population found in this study also suggests that the addition of an intron by mobile elements could provide an advantage to the integrated element itself, preventing further invasion by other elements.

The presence of region ‘a’ in the insertion sequence of JFF014_A09 supports the occurrence of a subsequent homologous recombination event after the integration of the mobile element. Because the sequence of region ‘a’ is found in six regions of the composite genome, we inferred that the ‘a’ sequence is a transposable element. We speculate that the homologous recombination that occurred among these homologous regions, one of which is located between the tRNA gene and a tRNA fragment sequence (e.g., JFF014_A09 in Figure 5), possibly created the split tRNA gene halves that are widely scattered in the genome (Figure 5).

Previous investigations of the sequence evolution of disrupted tRNA genes have focused on comparative genomics at the species level [13], [23], [32]. In this study, based on population genomics of a specific archaeal lineage within a subsurface microbial community, we have identified novel sequences that suggest that intron gain and the splitting of a tRNA gene have occurred within a species or sub-species. The unusually low diversity within this *Caldiarchaeum* community, in which *C. subterraneum* predominated, most likely resulted from a population bottleneck [33] when this facultatively aerobic microbial community arose at a mine site. Considering the extremely homogeneous genomic sequences of the genome fragments of *Caldiarchaeum* population predominated by *C. subterraneum*, the *C. subterraneum* community found in the microbial mat might have originated from one or a few cells, and the genomic diversity observed in this population is probably the result of this natural long-term continuous culture experiment. Metagenomic analysis targeting microbial communities produced by natural or artificial population bottlenecks should provide further information not only regarding the evolution of disrupted tRNAs, but also regarding other processes of genomic differentiation. Therefore, it should be very useful to collect as many metagenomic samples as possible from various microbial habitats to verify the proposed model of tRNA gene disruption.

### Conclusions

We identified three types of heterogeneity at the tRNA^Thr^(GGU) gene locus in a *Caldiarchaeum* population collected from a microbial mat isolated from a geothermal water stream of a sub-surface gold mine. We suggest that this heterogeneity could be generated by insertion of a transposable element and subsequent homologous recombination by the DNA recombinase encoded in this transposed element. Furthermore, the results also suggested that intron gain or loss by tRNA and the scattering of tRNA fragments to remote parts of the genome possibly occurred over a short time period within a single species, or among two extremely closely related species. This enabled us to propose a model to explain the evolution of tRNA.

## Materials and Methods

### Fosmid library, clones, and DNA sequences

The fosmid library was constructed from an unusual microbial community dominated by uncultured archaeotes, and the DNA of the archaeal genome fragments was sequenced as previously described [20], [21]. In brief, a metagenomic library consisting of 5,280 fosmid clones was constructed from high-molecular-weight DNA (fragments as large as 50 kbp), which was prepared from a microbial mat taken from a geothermal water stream at a depth of 320 m from the ground surface in a Japanese gold mine. All the fosmid clones in the library were extracted from an *Escherichia coli* culture, and the paired-end sequences of each cloned genomic fragment were sequenced. The putative archaeal genome fragments were then analysed using both a GS20 pyrosequencer and by Sanger sequencing. The composite circular genomic sequence of *C. subterraneum* (1,680,938 bp) was assembled from a set of 62 complete or partial fosmid sequences, and an additional 28 complete or partial fosmid sequences (including JFF006_G04) derived from *C. subterraneum* were also obtained [21]. In addition to the *C. subterraneum* SSU rRNA ribotype (19 clones), we found two ribotypes of an archaeal SSU rRNA gene in the library. Two clones were derived from *Caldiarchaeum* sp. (*Caldiarchaeum* ribotype II) and three clones were derived from *Nistrosocaldus* sp. [21]. The similarity of the SSU rRNA gene between the two *Caldiarchaeum* species was 96.6%.

In this study, we selected an additional fosmid clone, JFF014_A09, which is predicted to encode the tRNA^Thr^(GGU) gene locus of *C. subterraneum*, but consists of two genomic regions shown by paired-end-read information to be distantly located on the composite genome of *C. subterraneum*. Following the successful PCR amplification of tRNA^Thr^(GGU) using gene-specific primers, a partial sequence of clone JFF014_A09, including the boundary of the two genomic regions, was determined using an ABI 3100 DNA Sequencer (Applied Biosystems). The sequence was assembled by BLASTN analysis. We obtained at least two independent fosmid clones for each tRNA type, and sequence data for these clones has been deposited in the DDBJ/EMBL/GenBank database (Table S1).

We have a contract with a mining company about the sampling in a subsurface mine. The contract specifies that the name of the company cannot be disclosed when this manuscript is published.

### Prediction of tRNA genes and tRNA fragments

tRNA genes were predicted using tRNAscan-SE [34], with the Archaea-specific search mode and SPLITSX [6], using the following parameters: −p 0.55, −f 0, −h 3. The fragmented tRNA was subjected to a BLASTN search with the default parameters. The query term used was the sequence found in clone JFF006_G04 (that is, the sequence of the tRNA^Thr^(GGU) gene containing one intron).

### Characterization of the insertion sequence

An 813 bp ORF in clone JFF014_A09 (defined as region ‘b’) was predicted using the Glimmer program (http://www.ncbi.nlm.nih.gov/genomes/MICROBES/glimmer_3.cgi). Its protein function was predicted with SVMProt [24], a software program used extensively for the Support Vector Machine–mediated classification of proteins into functional families based on protein properties such as amino acid composition, hydrophobicity, charge, and secondary structure. A homology search of the 545-bp nucleotide sequence found in clone JFF014_A09 (defined as region ‘a’) against the composite genome of *C. subterraneum* was performed using BLASTN, with parameters −q −1, −X 100, −e 10^−100^, to identify pairwise matches with more than 80% coverage. The amino acid sequence of region ‘b’ was compared with all 1,793 proteins encoded in the composite genome of *C. subterraneum* using BLASTP, with the parameter −e 10^−20^ selected to identify pairwise matches with more than 80% coverage.

### Analysis of dinucleotide compositions

#### Measurement of dinucleotide compositional differences among DNA sequences

The average absolute dinucleotide relative abundance difference (δ*-value), which represents differences in dinucleotide compositions of two DNA sequences, was calculated as previously described [26]. Briefly, dinucleotide relative abundance values (ρ*_XY_) are defined as ρ*_XY_ = *f*_XY_/(*f*_X_×*f*_Y_), where *f*_X_ and *f*_XY_ denote the frequencies of the mononucleotide X and the dinucleotide XY, respectively. Both *f*_X_ and *f*_XY_ are computed from both the sense and reverse complement sequence. The δ*-value is given by δ*(f, g) = 1/16×∑ | ρ_XY_*(f) − ρ_XY_*(g)|×1000, where ρ_XY_*(f) and ρ_XY_*(g) are the abundance values calculated for the input sequences ‘f’ and ‘g’, respectively.

#### Data set

In total, 180 complete sequences, including 85 archaeal chromosomes and 95 archaeal plasmids, were downloaded from the NCBI web site (http://www.ncbi.nlm.nih.gov/sites/genome/) (September 2010). We selected 10 of the 95 archaeal plasmids as pNOB8-type-integrase-encoding plasmids based on a previous report [15]. Selection was based on two criteria. First, the plasmid needed to encode a tRNA fragment >30 bp long. Second, the tRNA gene needed to be immediately adjacent to a gene encoding a putative integrase similar to that encoded in the plasmid pNOB8 (NCBI Protein accession number YP_145765.1; BLASTP *E*-value<10^−10^). For the tRNA fragment search, all 3,742 archaeal tRNA gene sequences deposited in SPLITSdb [23] were used as query terms in a BLASTN analysis that used default parameters. The entire set of 180 DNA sequences (85 chromosomes, 10 plasmids encoding a pNOB8-type integrase, and 85 other plasmids) is shown in Table S2.

## Supporting Information

Figure S1 **Nucleotide sequence alignment of homologous ‘a’ regions in clone JFF014_A09 and the composite genomes of** ***C. subterraneum*** **.** A multiple sequence alignment of seven homologous ‘a’ regions was performed using ClustalW 2.0 [35] with the default parameters. Asterisks indicate conserved nucleotide positions. The designations ‘a’ and ‘a1–a6’ are as defined in Figure 3. The numbers in parentheses indicate positions in the composite genome of *C. subterraneum*.
(TIFF)

Figure S2 **Protein sequence alignment of the putative ORF encoded by region ‘b’ of clone JFF014_A09 and the composite genomes of** ***C. subterraneum*** **.** Amino acid sequences were aligned using ClustalW 2.0 [35] with the default parameters. Identical or similar amino acids are shown in the same colors. Asterisks indicate identical residues at that position. Partly conserved amino acids are indicated by dots, with two-dot regions having a higher degree of similarity than positions denoted with a single dot. The designations ‘b’ and ‘b1–b7’ are defined as in Figure 3. The gene ID for each ORF is shown in parentheses.
(TIFF)

Table S1 **Independent fosmid clones reveal the genomic heterogeneity of tRNA^Thr^ loci.** (*) See Figure 2 for types of tRNA^Thr^. A single gene ID in a column corresponds to a full nucleotide sequence or long partial nucleotide sequence of a fosmid clone. Two gene IDs in a column correspond to 5′ and 3′ end nucleotide sequences of a fosmid clone, respectively.
(DOC)

Table S2 Summary of δ*-values for 180 archaeal chromosomes and plasmids. ^a^Plasmids encoding pNOB8-type integrases are underlined.
(DOC)
