# Supplementary figures and images for: Genomic Heterogeneity in a Natural Archaeal Population Suggests a Model of tRNA Gene Disruption
Source: PLoS One. 2012 Mar 5;7(3):e32504. doi: 10.1371/journal.pone.0032504 (PMC3293823; doi:10.1371/journal.pone.0032504)

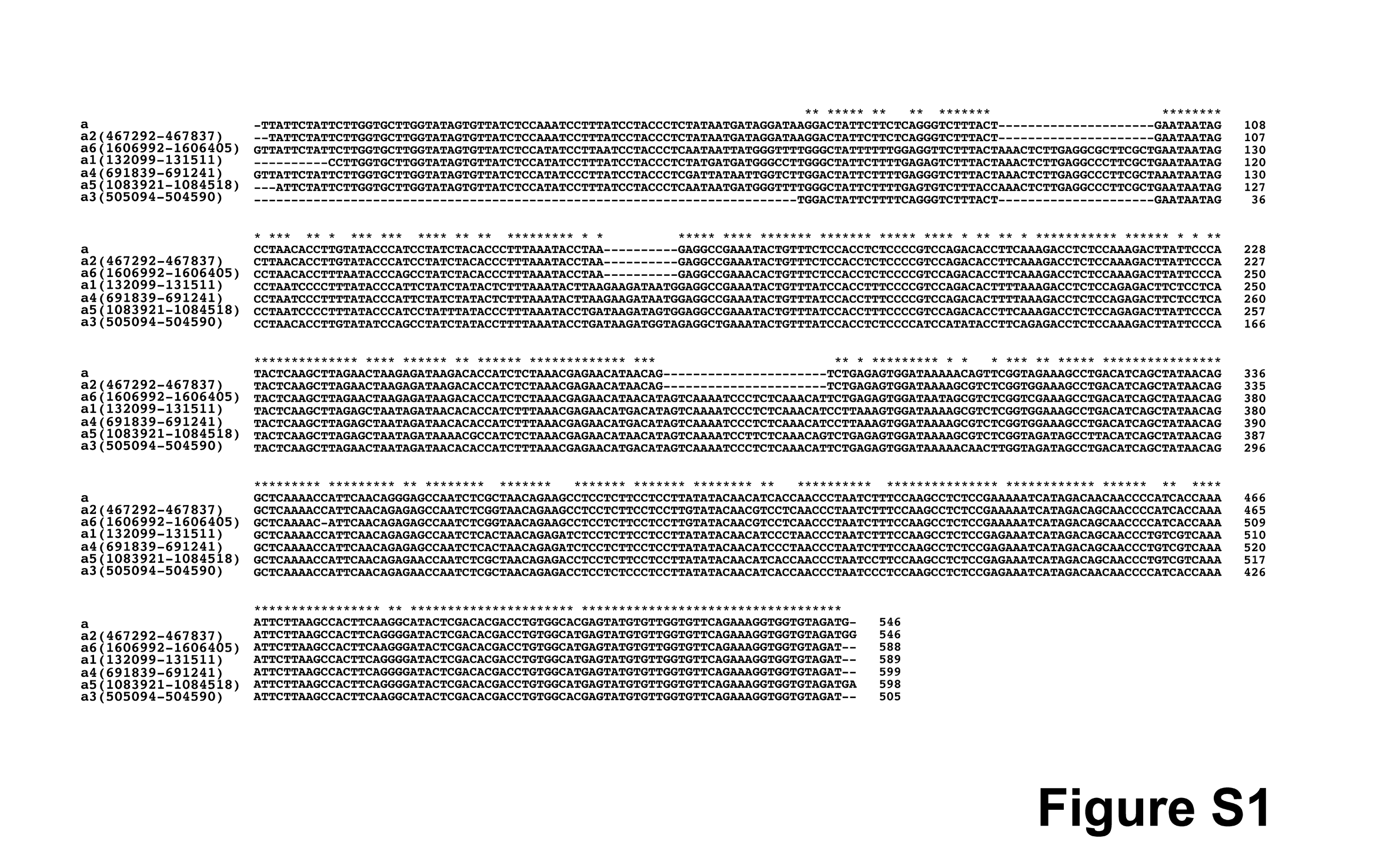

Supplement: Figure S1 — Nucleotide sequence alignment of homologous ‘a’ regions in clone JFF014_A09 and the composite genomes of C. subterraneum . A multiple sequence alignment of seven homologous ‘a’ regions was performed using ClustalW 2.0 [35] with the default parameters. Asterisks indicate conserved nucleotide positions. The designations ‘a’ and ‘a1–a6’ are as defined in Figure 3. The numbers in parentheses indicate positions in the composite genome of C. subterraneum. (TIFF) [file pone.0032504.s001.tiff]

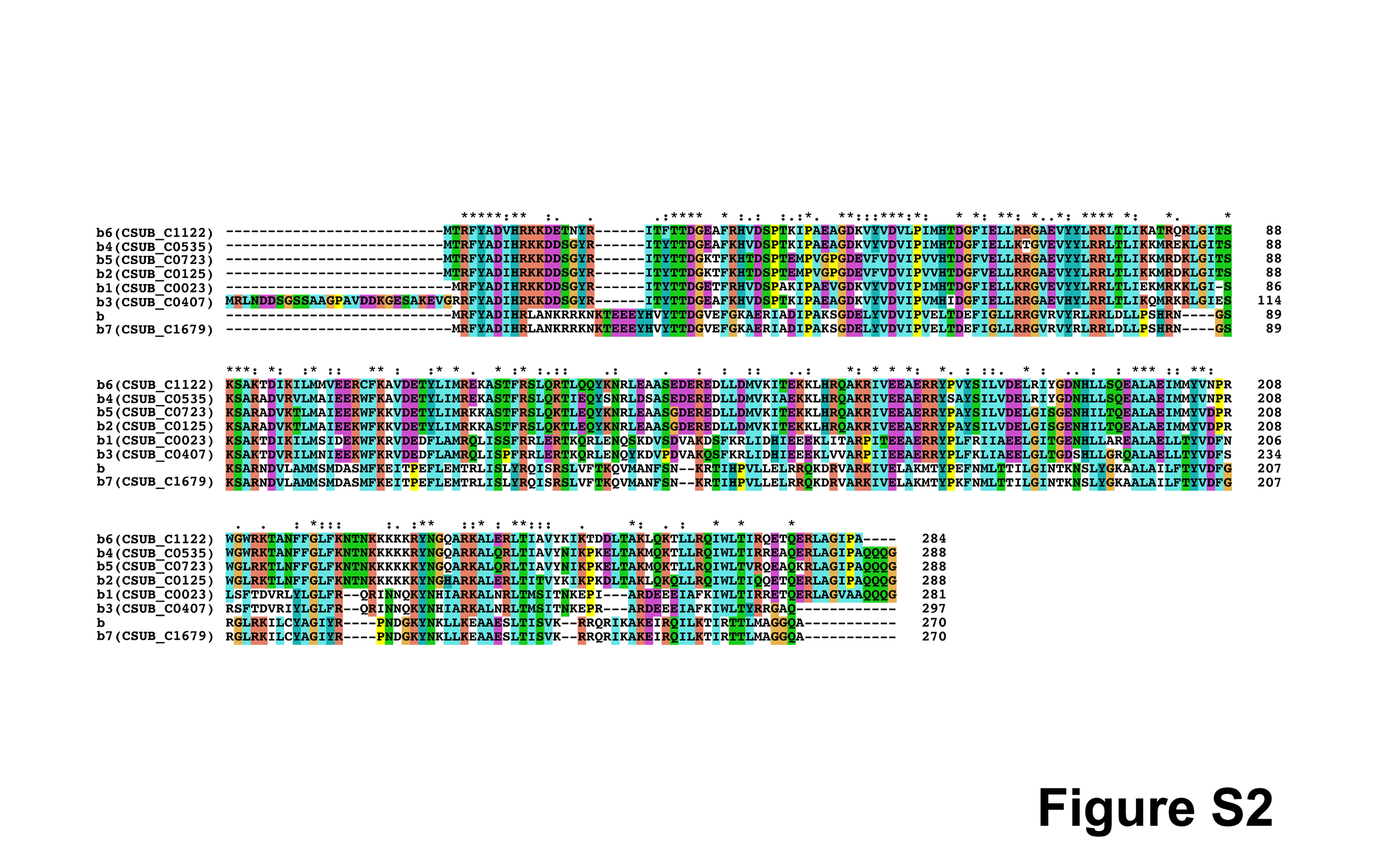

Supplement: Figure S2 — Protein sequence alignment of the putative ORF encoded by region ‘b’ of clone JFF014_A09 and the composite genomes of C. subterraneum . Amino acid sequences were aligned using ClustalW 2.0 [35] with the default parameters. Identical or similar amino acids are shown in the same colors. Asterisks indicate identical residues at that position. Partly conserved amino acids are indicated by dots, with two-dot regions having a higher degree of similarity than positions denoted with a single dot. The designations ‘b’ and ‘b1–b7’ are defined as in Figure 3. The gene ID for each ORF is shown in parentheses. (TIFF) [file pone.0032504.s002.tiff]
