# Supplementary material for: Genomic Heterogeneity in a Natural Archaeal Population Suggests a Model of tRNA Gene Disruption
Source: PLoS One. 2012 Mar 5;7(3):e32504. doi: 10.1371/journal.pone.0032504 (PMC3293823; doi:10.1371/journal.pone.0032504)
Supplement: Table S1 — Independent fosmid clones reveal the genomic heterogeneity of tRNAThr loci. (*) See Figure 2 for types of tRNAThr. A single gene ID in a column corresponds to a full nucleotide sequence or long partial nucleotide sequence of a fosmid clone. Two gene IDs in a column correspond to 5′ and 3′ end nucleotide sequences of a fosmid clone, respectively. (DOC) [file pone.0032504.s003.doc]

**Table S1. Independent fosmid clones reveal the genomic heterogeneity of tRNA^Thr^ loci.**

| **Type** (*) |  | **Fosmid Clone #** | **DDBJ/EMBL/GenBank ID** |
| --- | --- | --- | --- |
| A | (Thr-II) | JFF005_C07 | AG994435, AG994436 |
|  | (Thr-II) | JFF014_H10 | AG995507, AG995508 |
|  | (Thr-II) | JFF020_G01 | AP011902 |
|  | (Thr-II) | JFF049_D01 | AG998971, AG998972 |
|  | (Thr-F) | JFF001_D05 | AG993811, AG993812 |
|  | (Thr-F) | JFF006_H06 | AG994692, AG994693 |
|  | (Thr-F) | JFF008_H08 | AG994982, AG994983 |
|  | (Thr-F) | JFF033_F07 | AP011840 |
|  | (Thr-F) | JFF034_F07 | AG997646, AG997647 |
|  | (Thr-F) | JFF040_F10 | AG998269, AG998270 |
|  | (Thr-F) | JFF049_A03 | AG998933, AG998934 |
|  | (Thr-F) | JFF053_B02 | AG999415, AG999416 |
|  | (Thr-F) | JFF055_E03 | AG999651, AG999652 |
| B | (Thr-I) | JFF006_G04 | AP011657 |
|  | (Thr-I) | JFF026_E07 | AG996658 |
|  | (Thr-I) | JFF031_E12 | AG997258, AG997259 |
| C | (Thr-F & II) | JFF014_A09 | AP012034 |
|  | (Thr-F & II) | JFF028_E12 | AG996860, AG996861 |

(*) See Figure 2 for types of tRNA^Thr^. A single gene ID in a column corresponds to a full nucleotide sequence or long partial nucleotide sequence of a fosmid clone. Two gene IDs in a column correspond to 5’ and 3’ end nucleotide sequences of a fosmid clone, respectively.
