# Supplementary material for: Genomic Heterogeneity in a Natural Archaeal Population Suggests a Model of tRNA Gene Disruption
Source: PLoS One. 2012 Mar 5;7(3):e32504. doi: 10.1371/journal.pone.0032504 (PMC3293823; doi:10.1371/journal.pone.0032504)
Supplement: Table S2 — Summary of δ*-values for 180 archaeal chromosomes and plasmids. aPlasmids encoding pNOB8-type integrases are underlined. (DOC) [file pone.0032504.s004.doc]

**Table S2. Summary of δ*-values for 180 archaeal chromosomes and plasmids**

| **Rank** | **δ*-value** | **Definition^a^** | **Genome size (bp)** | **Accession** |
| --- | --- | --- | --- | --- |
| 1 | 25.768 | *Sulfolobus tengchongensis* plasmid pTC | 20,417 | NC_005969 |
| 2 | 32.563 | *Pyrococcus abyssi* GE5 plasmid pGT5 | 3,444 | NC_001773 |
| 3 | 44.084 | *Metallosphaera sedula* DSM 5348 chromosome | 2,191,517 | NC_009440 |
| 4 | 45.774 | *Sulfolobus acidocaldarius* DSM 639 chromosome | 2,225,959 | NC_007181 |
| 5 | 51.957 | *Sulfolobus islandicus* L.D.8.5 plasmid pLD8501 | 26,615 | NC_013770 |
| 6 | 52.274 | *Sulfolobus islandicus* plasmid pSOG2 | 25,960 | NC_010598 |
| 7 | 53.443 | *Sulfolobus islandicus* Y.N.15.51 plasmid pYN01 | 42,245 | NC_012624 |
| 8 | 55.095 | *Sulfolobus* sp. NOB8H2 plasmid pNOB8 | 41,229 | NC_006493 |
| 9 | 57.028 | *Sulfolobus islandicus* REN1H1 plasmid pRN1 | 5,350 | NC_001771 |
| 10 | 57.881 | *Acidianus hospitalis* plasmid pAH1 | 28,649 | NC_011299 |
| 11 | 59.013 | *Thermosphaera aggregans* DSM 11486 chromosome | 1,316,595 | NC_014160 |
| 12 | 60.737 | *Sulfolobus islandicus* plasmid pING1 | 24,554 | NC_004852 |
| 13 | 60.871 | *Pyrococcus abyssi* GE5 chromosome | 1,765,118 | NC_000868 |
| 14 | 61.101 | *Sulfolobus islandicus* plasmid pSOG1 | 29,000 | NC_010597 |
| 15 | 61.635 | *Sulfolobus islandicus* plasmid pHVE14 | 35,422 | NC_006425 |
| 16 | 64.593 | *Sulfolobus islandicus* plasmid pARN4 | 26,476 | NC_006424 |
| 17 | 64.832 | *Sulfolobus solfataricus* P2 chromosome | 2,992,245 | NC_002754 |
| 18 | 64.971 | *Sulfolobus tokodaii* str. 7 chromosome | 2,694,756 | NC_003106 |
| 19 | 65.084 | *Sulfolobus islandicus* Y.G.57.14 chromosome | 2,702,058 | NC_012622 |
| 20 | 65.447 | *Pyrococcus furiosus* DSM 3638 | 1,908,256 | NC_003413 |
| 21 | 65.534 | *Pyrococcus horikoshii* OT3 | 1,738,505 | NC_000961 |
| 22 | 65.971 | *Sulfolobus islandicus* M.16.4 chromosome | 2,586,647 | NC_012726 |
| 23 | 66.135 | *Sulfolobus islandicus* Y.N.15.51 chromosome | 2,812,165 | NC_012623 |
| 24 | 66.257 | *Sulfolobus islandicus* M.16.27 chromosome | 2,692,402 | NC_012632 |
| 25 | 66.559 | *Sulfolobus islandicus* L.D.8.5 chromosome | 2,722,032 | NC_013769 |
| 26 | 66.771 | *Sulfolobus islandicus* L.S.2.15 chromosome | 2,736,272 | NC_012589 |
| 27 | 67.138 | *Sulfolobus islandicus* M.14.25 chromosome | 2,608,832 | NC_012588 |
| 28 | 69.291 | *Sulfolobus islandicus* plasmid pKEF9 | 28,930 | NC_006422 |
| 29 | 70.964 | *Staphylothermus marinus* F1 chromosome | 1,570,485 | NC_009033 |
| 30 | 70.992 | *Sulfolobus islandicus* plasmid pARN3 | 26,200 | NC_006423 |
| 31 | 71.581 | *Methanosarcina barkeri* str. *fusaro* plasmid 1 | 36,358 | NC_007349 |
| 32 | 71.765 | *Archaeoglobus profundus* DSM 5631 | 1,560,622 | NC_013741 |
| 33 | 71.995 | *Acidilobus saccharovorans* 345-15 chromosome | 1,496,453 | NC_014374 |
| 34 | 78.173 | *Sulfolobus islandicus* plasmid pXZ1 | 6,970 | NC_010365 |
| 35 | 80.669 | *Thermococcus onnurineus* NA1 | 1,847,607 | NC_011529 |
| 36 | 81.275 | *Staphylothermus hellenicus* DSM 12710 chromosome | 1,580,347 | NC_014205 |
| 37 | 81.557 | *Aeropyrum pernix* K1 | 1,669,696 | NC_000854 |
| 38 | 83.495 | *Sulfolobus islandicus* HEN7H2 plasmid pHEN7 | 7,830 | NC_004853 |
| 39 | 84.328 | *Thermococcus sibiricus* MM 739 | 1,845,800 | NC_012883 |
| 40 | 84.949 | *Thermoproteus neutrophilus* V24Sta chromosome | 1,769,823 | NC_010525 |
| 41 | 85.567 | *Desulfurococcus kamchatkensis* 1221n chromosome | 1,365,223 | NC_011766 |
| 42 | 85.902 | *Archaeoglobus profundus* DSM 5631 plasmid pArcpr01 | 2,801 | NC_013742 |
| 43 | 86.262 | *Archaeoglobus profundus* plasmid pGS5 | 2,802 | NC_012890 |
| 44 | 86.426 | *Thermoplasma volcanium* GSS1 chromosome | 1,584,804 | NC_002689 |
| 45 | 88.976 | *Thermococcus kodakarensis* KOD1 | 2,088,737 | NC_006624 |
| 46 | 89.238 | *Pyrococcus* sp. 12/1 plasmid pP12-1 | 12,205 | NC_014110 |
| 47 | 92.028 | *Thermococcus* sp. AMT11 plasmid pAMT11 | 20,534 | NC_013177 |
| 48 | 94.011 | *Sulfolobus islandicus* REN1H1 plasmid pRN2 | 6,959 | NC_002101 |
| 49 | 94.999 | *Methanohalophilus mahii* plasmid pML | 2,158 | NC_005563 |
| 50 | 96.126 | *Ignicoccus hospitalis* KIN4/I chromosome | 1,297,538 | NC_009776 |
| 51 | 96.184 | *Thermococcus nautilus* plasmid pTN2 | 13,015 | NC_014115 |
| 52 | 97.705 | *Ignisphaera aggregans* DSM 17230 chromosome | 1,875,953 | NC_014471 |
| 53 | 99.497 | *Sulfolobus neozealandicus* plasmid pORA1 | 9,689 | NC_006906 |
| 54 | 99.650 | *Acidianus ambivalens* Lei 10 plasmid pDL10 | 7,598 | NC_005562 |
| 55 | 101.761 | *Methanosarcina mazei* Go1 chromosome | 4,096,345 | NC_003901 |
| 56 | 102.363 | *Methanosarcina acetivorans* C2A chromosome | 5,751,492 | NC_003552 |
| 57 | 102.646 | *Sulfolobus solfataricus* IT3 plasmid pIT3 | 4,967 | NC_005907 |
| 58 | 104.169 | *Methanococcoides burtonii* DSM 6242 | 2,575,032 | NC_007955 |
| 59 | 104.351 | *Archaeoglobus fulgidus* DSM 4304 | 2,178,400 | NC_000917 |
| 60 | 106.123 | *Pyrobaculum calidifontis* JCM 11548 chromosome | 2,009,313 | NC_009073 |
| 61 | 106.146 | *Thermococcus gammatolerans* EJ3 | 2,045,438 | NC_012804 |
| 62 | 106.391 | Uncultured methanogenic archaeon RC-I chromosome | 3,179,916 | NC_009464 |
| 63 | 108.010 | *Sulfolobus islandicus* plasmid pSSVx | 5,705 | NC_010011 |
| 64 | 108.379 | *Methanothermobacter thermautotrophicus* Z-245 plasmid pFZ1 | 11,014 | NC_001337 |
| 65 | 108.476 | *Thermococcus* sp. 26/2 plasmid pT26-2 | 21,566 | NC_014116 |
| 66 | 108.831 | *Methanocaldococcus vulcanius* M7 plasmid pMETVU02 | 4,704 | NC_013409 |
| 67 | 109.634 | *Pyrobaculum arsenaticum* DSM 13514 | 2,121,076 | NC_009376 |
| 68 | 109.742 | *Pyrobaculum islandicum* DSM 4184 chromosome | 1,826,402 | NC_008701 |
| 69 | 110.149 | *Methanocaldococcus vulcanius* M7 | 1,746,329 | NC_013407 |
| 70 | 110.279 | *Methanocaldococcus jannaschii* DSM 2661 plasmid small ECE | 16,550 | NC_001733 |
| 71 | 110.322 | *Thermococcus nautilus* 30-1 plasmid pTN1 | 3,619 | NC_009658 |
| 72 | 112.288 | *Aciduliprofundum boonei* T469 chromosome | 1,486,778 | NC_013926 |
| 73 | 115.009 | *Methanocaldococcus jannaschii* DSM 2661 plasmid large ECE | 58,407 | NC_001732 |
| 74 | 115.091 | *Methanothermobacter thermautotrophicus* THF plasmid pFV1 | 13,514 | NC_001336 |
| 75 | 116.438 | *Methanohalobium evestigatum* Z-7303 plasmid pMETEV01 | 163,915 | NC_014254 |
| 76 | 119.835 | *Thermofilum pendens* Hrk 5 chromosome | 1,781,889 | NC_008698 |
| 77 | 120.212 | *Methanohalobium evestigatum* Z-7303 chromosome | 2,242,317 | NC_014253 |
| 78 | 123.232 | *Methanosarcina acetivorans* C2A plasmid pC2A | 5,467 | NC_002097 |
| 79 | 127.446 | *Methanobrevibacter ruminantium* M1 chromosome | 2,937,203 | NC_013790 |
| 80 | 128.569 | *Thermoplasma acidophilum* DSM 1728 chromosome | 1,564,906 | NC_002578 |
| 81 | 128.813 | *Methanohalophilus mahii* DSM 5219 chromosome | 2,012,424 | NC_014002 |
| 82 | 129.864 | *Hyperthermus butylicus* DSM 5456 chromosome | 1,667,163 | NC_008818 |
| 83 | 132.187 | *Methanocaldococcus fervens* AG86 plasmid pMEFER01 | 22,190 | NC_013157 |
| 84 | 132.308 | *Halalkalicoccus jeotgali* B3 plasmid 5 | 23,727 | NC_014302 |
| 85 | 132.813 | *Methanospirillum hungatei* JF-1 | 3,544,738 | NC_007796 |
| 86 | 133.008 | *Methanosphaera stadtmanae* DSM 3091 chromosome | 1,767,403 | NC_007681 |
| 87 | 133.161 | *Methanocaldococcus* sp. FS406-22 chromosome | 1,760,939 | NC_013887 |
| 88 | 134.024 | *Thermofilum pendens* Hrk 5 plasmid pTPEN01 | 31,504 | NC_008696 |
| 89 | 135.683 | *Methanocaldococcus jannaschii* DSM 2661 chromosome | 1,664,970 | NC_000909 |
| 90 | 137.358 | *Methanobrevibacter smithii* ATCC 35061 chromosome | 1,853,160 | NC_009515 |
| 91 | 139.451 | *Methanocaldococcus infernus* ME chromosome | 1,328,194 | NC_014122 |
| 92 | 139.489 | *Pyrobaculum aerophilum* str. IM2 chromosome | 2,222,430 | NC_003364 |
| 93 | 139.565 | Candidatus *Methanoregula boonei* 6A8 chromosome | 2,542,943 | NC_009712 |
| 94 | 144.503 | *Methanocaldococcus fervens* AG86 | 1,485,061 | NC_013156 |
| 95 | 144.700 | *Thermoplasma acidophilum* plasmid pTA1 | 15,723 | NC_008318 |
| 96 | 144.992 | *Vulcanisaeta distributa* DSM 14429 chromosome | 2,374,137 | NC_014537 |
| 97 | 145.025 | *Methanocella paludicola* SANAE chromosome | 2,957,635 | NC_013665 |
| 98 | 148.499 | *Methanoplanus petrolearius* DSM 11571 chromosome | 2,843,290 | NC_014507 |
| 99 | 149.293 | *Pyrococcus* sp. JT1 plasmid pRT1 | 3,373 | NC_003026 |
| 100 | 149.971 | *Methanococcus maripaludis* C6 | 1,744,193 | NC_009975 |
| 101 | 150.919 | *Methanocaldococcus* sp. FS406-22 plasmid pFS01 | 12,197 | NC_013888 |
| 102 | 151.871 | *Methanococcus maripaludis* C7 | 1,772,694 | NC_009637 |
| 103 | 153.760 | *Methanococcus maripaludis* C5 | 1,780,761 | NC_009135 |
| 104 | 154.307 | *Haloarcula* sp. AS7094 plasmid pSCM201 | 3,463 | NC_006426 |
| 105 | 154.601 | *Ferroglobus placidus* DSM 10642 chromosome | 2,196,266 | NC_013849 |
| 106 | 157.100 | *Methanococcus maripaludis* S2 | 1,661,137 | NC_005791 |
| 107 | 158.222 | *Caldivirga maquilingensis* IC-167 chromosome | 2,077,567 | NC_009954 |
| 108 | 160.671 | Haloarchaeal coccus LOC-1 GN101 plasmid pHGN1 | 1,765 | NC_002124 |
| 109 | 162.373 | *Haloarcula marismortui* ATCC 43049 plasmid pNG100 | 33,303 | NC_006389 |
| 110 | 165.088 | *Methanothermobacter thermautotrophicus* Marburg plasmid pME2001 | 4,439 | NC_002125 |
| 111 | 165.589 | *Methanothermobacter marburgensis* str. Marburg plasmid pMTBMA4 | 4,440 | NC_014409 |
| 112 | 165.864 | Candidatus *Methanosphaerula palustris* E1-9c | 2,922,917 | NC_011832 |
| 113 | 168.312 | *Methanococcus voltae* A3 chromosome | 1,936,387 | NC_014222 |
| 114 | 169.113 | *Methanococcus vannielii* SB | 1,720,048 | NC_009634 |
| 115 | 170.598 | *Methanocaldococcus vulcanius* M7 plasmid pMETVU01 | 10,704 | NC_013408 |
| 116 | 171.541 | *Methanocorpusculum labreanum* Z chromosome | 1,804,962 | NC_008942 |
| 117 | 176.271 | *Haloarcula marismortui* ATCC 43049 plasmid pNG500 | 132,678 | NC_006393 |
| 118 | 178.267 | *Methanosaeta thermophila* PT chromosome | 1,879,471 | NC_008553 |
| 119 | 182.032 | *Nanoarchaeum equitans* Kin4-M chromosome | 490,885 | NC_005213 |
| 120 | 186.010 | *Haloarcula marismortui* ATCC 43049 plasmid pNG200 | 33,452 | NC_006390 |
| 121 | 186.784 | *Picrophilus torridus* DSM 9790 chromosome | 1,545,895 | NC_005877 |
| 122 | 188.726 | *Methanococcus maripaludis* C5 plasmid pURB500 | 8,285 | NC_001811 |
| 123 | 188.759 | *Methanococcus maripaludis* C5 plasmid pMMC501 | 8,285 | NC_009136 |
| 124 | 191.526 | *Halalkalicoccus jeotgali* B3 plasmid 2 | 363,534 | NC_014299 |
| 125 | 194.003 | *Methanothermobacter thermautotrophicus* plasmid pME2200 | 6,205 | NC_000905 |
| 126 | 194.979 | *Haloarcula marismortui* ATCC 43049 plasmid pNG400 | 50,060 | NC_006392 |
| 127 | 195.077 | *Halalkalicoccus jeotgali* B3 plasmid 6 | 6,951 | NC_014303 |
| 128 | 196.534 | *Methanoculleus marisnigri* JR1 chromosome | 2,478,101 | NC_009051 |
| 129 | 197.665 | *Haloterrigena turkmenica* DSM 5511 plasmid pHTUR03 | 180,781 | NC_013746 |
| 130 | 197.676 | *Methanopyrus kandleri* AV19 | 1,694,969 | NC_003551 |
| 131 | 198.366 | *Natronomonas pharaonis* DSM 2160 plasmid PL131 | 130,989 | NC_007427 |
| 132 | 200.197 | *Halobacterium salinarum* R1 plasmid PHS4 | 40,894 | NC_010367 |
| 133 | 201.514 | *Natronobacterium* sp. AS-7091 plasmid pNB101 | 2,538 | NC_005239 |
| 134 | 204.129 | *Methanococcus aeolicus* Nankai-3 | 1,569,500 | NC_009635 |
| 135 | 205.358 | *Halorubrum lacusprofundi* ATCC 49239 plasmid pHLAC01 | 431,338 | NC_012030 |
| 136 | 205.600 | *Haloarcula marismortui* ATCC 43049 plasmid pNG700 | 410,554 | NC_006395 |
| 137 | 205.602 | *Haloarcula marismortui* ATCC 43049 plasmid pNG300 | 39,521 | NC_006391 |
| 138 | 205.908 | *Haloferax volcanii* DS2 plasmid pHV1 | 85,092 | NC_013968 |
| 139 | 207.519 | *Halalkalicoccus jeotgali* B3 plasmid 4 | 44,459 | NC_014301 |
| 140 | 211.338 | *Halalkalicoccus jeotgali* B3 plasmid 1 | 406,285 | NC_014298 |
| 141 | 211.678 | *Halobacterium salinarum* R1 plasmid PHS1 | 147,625 | NC_010366 |
| 142 | 211.849 | *Haloterrigena turkmenica* DSM 5511 plasmid pHTUR06 | 15,815 | NC_013749 |
| 143 | 212.081 | *Haloquadratum walsbyi* DSM 16790 plasmid PL47 | 46,867 | NC_008213 |
| 144 | 213.293 | *Halobacterium salinarum* plasmid pHSB | 1,736 | NC_002121 |
| 145 | 214.027 | *Natrinema* sp. CX2021 plasmid pZMX201 | 1,668 | NC_006996 |
| 146 | 214.067 | *Halobacterium* sp. NRC-1 plasmid pNRC100 | 191,346 | NC_001869 |
| 147 | 214.896 | *Haloquadratum walsbyi* DSM 16790 | 3,132,494 | NC_008212 |
| 148 | 220.339 | *Halobacterium* sp. NRC-1 plasmid pNRC200 | 365,425 | NC_002608 |
| 149 | 221.020 | *Natronomonas pharaonis* DSM 2160 plasmid PL23 | 23,486 | NC_007428 |
| 150 | 221.460 | *Halobacterium salinarum* R1 plasmid PHS2 | 194,963 | NC_010369 |
| 151 | 221.797 | *Halorubrum lacusprofundi* ATCC 49239 chromosome 2 | 525,943 | NC_012028 |
| 152 | 224.810 | *Halobacterium salinarum* R1 plasmid PHS3 | 284,332 | NC_010368 |
| 153 | 229.263 | *Natrialba magadii* ATCC 43099 plasmid pNMAG02 | 254,950 | NC_013924 |
| 154 | 231.459 | *Halalkalicoccus jeotgali* B3 plasmid 3 | 44,576 | NC_014300 |
| 155 | 231.933 | *Haloarcula marismortui* ATCC 43049 plasmid pNG600 | 155,300 | NC_006394 |
| 156 | 245.800 | *Natronomonas pharaonis* DSM 2160 | 2,595,221 | NC_007426 |
| 157 | 247.725 | *Halorubrum saccharovorum* plasmid pZMX101 | 3,918 | NC_004531 |
| 158 | 252.378 | *Haloferax volcanii* DS2 plasmid pHV4 | 635,786 | NC_013966 |
| 159 | 253.712 | *Haloterrigena turkmenica* DSM 5511 plasmid pHTUR05 | 71,062 | NC_013748 |
| 160 | 255.258 | *Haloferax volcanii* DS2 plasmid pHV2 | 6,359 | NC_013965 |
| 161 | 257.697 | *Haloterrigena thermotolerans* plasmid pSN | 2,272 | NC_013536 |
| 162 | 262.067 | *Natrialba magadii* ATCC 43099 plasmid pNMAG01 | 378,348 | NC_013923 |
| 163 | 266.075 | *Natrialba magadii* ATCC 43099 chromosome | 3,751,858 | NC_013922 |
| 164 | 268.352 | *Haloterrigena turkmenica* DSM 5511 plasmid pHTUR02 | 413,648 | NC_013745 |
| 165 | 269.746 | *Halobacterium* sp. NRC-1 chromosome | 2,014,239 | NC_002607 |
| 166 | 270.993 | *Halobacterium salinarum* R1 | 2,000,962 | NC_010364 |
| 167 | 271.262 | *Halobacterium salinarum* plasmid pPHIHL | 12,041 | NC_010088 |
| 168 | 272.364 | *Sulfolobus solfataricus* P2 plasmid pSSVi | 5,740 | NC_013777 |
| 169 | 279.694 | *Halorhabdus utahensis* DSM 12940 chromosome | 3,116,795 | NC_013158 |
| 170 | 282.229 | *Halalkalicoccus jeotgali* B3 chromosome | 2,809,118 | NC_014297 |
| 171 | 284.990 | *Halobacterium salinarum* plasmid pHH205 | 16,341 | NC_003158 |
| 172 | 290.030 | *Halomicrobium mukohataei* DSM 12286 plasmid pHmuk01 | 221,862 | NC_013201 |
| 173 | 294.587 | *Haloferax volcanii* DS2 plasmid pHV3 | 437,906 | NC_013964 |
| 174 | 295.716 | *Haloterrigena turkmenica* DSM 5511 plasmid pHTUR01 | 698,495 | NC_013744 |
| 175 | 295.973 | *Halomicrobium mukohataei* DSM 12286 | 3,110,487 | NC_013202 |
| 176 | 296.221 | *Haloferax volcanii* DS2 chromosome | 2,847,757 | NC_013967 |
| 177 | 300.721 | *Natrialba magadii* ATCC 43099 plasmid pNMAG03 | 58,487 | NC_013925 |
| 178 | 313.447 | *Halorubrum lacusprofundi* ATCC 49239 chromosome 1 | 2,735,295 | NC_012029 |
| 179 | 319.692 | *Haloterrigena turkmenica* DSM 5511 plasmid pHTUR04 | 171,943 | NC_013747 |
| 180 | 323.909 | *Haloterrigena turkmenica* DSM 5511 | 3,889,038 | NC_013743 |

^a^Plasmids encoding pNOB8-type integrases are underlined.
